# Comparative Accuracy of Diagnosis by Collective Intelligence of Multiple Physicians vs Individual Physicians

**DOI:** 10.1001/jamanetworkopen.2019.0096

**Published:** 2019-03-01

**Authors:** Michael L. Barnett, Dhruv Boddupalli, Shantanu Nundy, David W. Bates

**Affiliations:** 1 Department of Health Policy and Management, Harvard T.H. Chan School of Public Health, Boston, Massachusetts; 2 Division of General Internal Medicine and Primary Care, Department of Medicine, Brigham and Women’s Hospital, Boston, Massachusetts; 3 Department of Medicine, Harvard Medical School, Boston, Massachusetts; 4 Department of Medicine, University of California, San Francisco; 5 Milken Institute School of Public Health, George Washington University, Washington, DC

## Abstract

**Importance:**

The traditional approach of diagnosis by individual physicians has a high rate of misdiagnosis. Pooling multiple physicians’ diagnoses (collective intelligence) is a promising approach to reducing misdiagnoses, but its accuracy in clinical cases is unknown to date.

**Objective:**

To assess how the diagnostic accuracy of groups of physicians and trainees compares with the diagnostic accuracy of individual physicians.

**Design, Setting, and Participants:**

Cross-sectional study using data from the Human Diagnosis Project (Human Dx), a multicountry data set of ranked differential diagnoses by individual physicians, graduate trainees, and medical students (users) solving user-submitted, structured clinical cases. From May 7, 2014, to October 5, 2016, groups of 2 to 9 randomly selected physicians solved individual cases. Data analysis was performed from March 16, 2017, to July 30, 2018.

**Main Outcomes and Measures:**

The primary outcome was diagnostic accuracy, assessed as a correct diagnosis in the top 3 ranked diagnoses for an individual; for groups, the top 3 diagnoses were a collective differential generated using a weighted combination of user diagnoses with a variety of approaches. A version of the McNemar test was used to account for clustering across repeated solvers to compare diagnostic accuracy.

**Results:**

Of the 2069 users solving 1572 cases from the Human Dx data set, 1228 (59.4%) were residents or fellows, 431 (20.8%) were attending physicians, and 410 (19.8%) were medical students. Collective intelligence was associated with increasing diagnostic accuracy, from 62.5% (95% CI, 60.1%-64.9%) for individual physicians up to 85.6% (95% CI, 83.9%-87.4%) for groups of 9 (23.0% difference; 95% CI, 14.9%-31.2%; *P* < .001). The range of improvement varied by the specifications used for combining groups’ diagnoses, but groups consistently outperformed individuals regardless of approach. Absolute improvement in accuracy from individuals to groups of 9 varied by presenting symptom from an increase of 17.3% (95% CI, 6.4%-28.2%; *P* = .002) for abdominal pain to 29.8% (95% CI, 3.7%-55.8%; *P* = .02) for fever. Groups from 2 users (77.7% accuracy; 95% CI, 70.1%-84.6%) to 9 users (85.5% accuracy; 95% CI, 75.1%-95.9%) outperformed individual specialists in their subspecialty (66.3% accuracy; 95% CI, 59.1%-73.5%; *P* < .001 vs groups of 2 and 9).

**Conclusions and Relevance:**

A collective intelligence approach was associated with higher diagnostic accuracy compared with individuals, including individual specialists whose expertise matched the case diagnosis, across a range of medical cases. Given the few proven strategies to address misdiagnosis, this technique merits further study in clinical settings.

## Key Points

**Question:**

Is a collective intelligence approach of pooling multiple clinician and medical student diagnoses associated with improvement in diagnostic accuracy in online, structured clinical cases?

**Findings:**

This cross-sectional study analyzing data from the Human Diagnosis Project found that, across a broad range of medical cases and common presenting symptoms, independent differential diagnoses of multiple physicians combined into a weighted list significantly outperformed diagnoses of individual physicians with groups as small as 2, and accuracy increased with larger groups up to 9 physicians. Groups of nonspecialists also significantly outperformed individual specialists solving cases matched to the individual specialist’s specialty.

**Meaning:**

Pooling the diagnoses of multiple physicians into a ranked list could be an effective approach to improving diagnostic accuracy, but further study in a clinical setting is needed.

## Teaser

This cross-sectional study uses cases from the Human Diagnosis Project to compare the accuracy of single-physicians’ diagnoses with pooled diagnoses from groups of physicians.

## Introduction

Diagnosis is central to the practice of medicine, yet studies suggest that misdiagnosis is prevalent even for common conditions with potential morbidity and mortality.^1,2,3,4,5,6,7^ For centuries, the prevailing model of diagnosis has been an individual practitioner assessing the patient and arriving at a diagnostic strategy on his or her own. One frequent exception, however, is in hospitals where team rounds, case conferences, and tumor boards are routinely used. Collaborative, team-based diagnosis is thought to be superior to individual diagnosis and has been championed by the National Academy of Medicine as a means to reduce diagnostic errors.^5^

Despite the favorable view of team-based diagnosis, there is little evidence on the relative benefit of group-based approaches to diagnosis in improving diagnostic accuracy.^5^ The ability of collective intelligence—groups of individuals acting independently or collectively—to outperform individuals acting alone has gained prominence in politics, business, and economics, with studies identifying conditions in which collective intelligence appears beneficial.^8,9,10,11,12,13,14,15^ Collective intelligence facilitated by software is particularly well suited for medicine because it may offer superior performance with little coordination.

Previous studies of collective intelligence in medicine focused mainly on well-defined tasks and usually assessed binary decisions, such as interpretation of mammography and skin lesions.^8,9,10,11^ To date, the existing evidence on the role of collective intelligence in general clinical diagnosis has been limited to single-center studies of medical students.^11,16^ However, in most medical specialties, the diagnostic process requires combining many pieces of information while considering multiple diagnoses. The present study focused on general clinical diagnosis rather than binary decisions in narrow medical domains and, to our knowledge, is the largest study to date of collective intelligence in medicine.

The Human Diagnosis Project (Human Dx) is a multinational medical project in which physicians and medical students solve teaching cases. We used teaching cases from Human Dx to study whether collective intelligence is associated with improvement in diagnostic accuracy in hundreds of clinical cases across multiple specialties and how group performance compared with the performance of individual specialists whose expertise matched the case diagnosis.

## Methods

### Case Platform

The Human Dx online platform is designed for attending physicians and fellows, residents, and medical students (users) to practice authoring and diagnosing teaching cases. Human Dx users create teaching cases from their own clinical practice with key elements of the history, physical, and diagnostic tests (eg, laboratory and imaging studies) and identify the intended diagnosis or the differential diagnoses. Respondents independently generate ranked differential diagnoses for a case and are notified if they are correct or incorrect. Cases are rated by respondents for clarity and teaching value. Cases that are tagged by multiple users as having problems with clarity or quality are removed from the system or revised. Human Dx tags each case with multiple specialties based on both the intended diagnosis and top differential diagnoses generated by the respondents. As of December 28, 2018, more than 14 000 users of all specialties from more than 80 countries have made more than 230 000 contributions authoring and diagnosing cases. The study was deemed not human subjects research and therefore exempt from review by Harvard’s Institutional Review Board and followed the Strengthening the Reporting of Observational Studies in Epidemiology (STROBE) reporting guideline.

### Study Sample

We analyzed all cases authored by users between May 7, 2014, through October 5, 2016, with 10 or more respondents (1572 cases). We captured a sample of cases with 10 or more respondents, dropping the first solve attempt in the Human Dx system by any user to account for the learning curve in using the platform. We used self-reported Human Dx profiles to categorize each user by specialty into internal medicine (general internal medicine and its subspecialties), surgery (general surgery and surgical subspecialties), other specialties (pediatrics, anesthesiology, and all other specialties), and medical student. Users of all specialties from the United States and 46 other countries were included in the analysis.

For each case, we randomly sampled 10 solve attempts (a user can only submit 1 solve attempt per case). Our analysis included users who attempted more than 1 case.

### Scoring Solve Attempts

The Human Dx system allows respondents to input diagnoses from an existing list of terms or as free text. In the base case specification (see below for more details), an individual solve attempt was marked correct if any of the top 3 diagnoses from the respondent matched the case author’s intended diagnosis or differential diagnoses. There can be ambiguity in deciding whether an intended diagnosis is equivalent to a respondent’s diagnoses regardless of the guidelines provided to reviewers. For example, 2 users may disagree on whether the diagnoses of hyperparathyroidism and adenoma parathyroid both count as correct for a given case. Because of this issue, we assessed interrater reliability for adjudicating correct solves by having 2 of us (D.B. and S.N.) use prespecified guidelines to manually compare the respondents’ diagnoses with the author’s intended diagnosis or differential diagnoses for sets of 100 cases using iterative consensus to refine guidelines. Across test cases, interrater agreement was good (Cohen κ = 0.70). For the study sample, all solve attempts were manually scored by one of us with internal medicine training (D.B.).

### Collective Intelligence Rule: Proportional Weighting

To create a differential diagnosis informed by the collective contributions of multiple Human Dx users, we generated a collective differential with a set of rules. In the base case used as the default for most of the analyses, we used a 1/*n* weighting rule. For an individual solver with *n* diagnoses in his or her differential diagnoses, we weighted each diagnosis by its order in the differential (ie, the *n*th diagnosis would be weighted 1/*n*) to downweight diagnoses with lower ranks in long lists. For each group of users ranging from 2 to 9 members, the weights of each diagnosis among solvers were summed to produce a ranked list of diagnoses: the collective differential. In the base case, we used the top 3 diagnoses in this collective differential to adjudicate the differential as correct if the intended diagnosis or differential diagnosis was included in the top 3 collective differential diagnoses.

We tested the performance of our collective differential approach using a range of variations on the base case rules above across 3 dimensions: the weighting rule for creating collective differentials, the number of diagnoses in the collective differential used to adjudicate success, and the number of user diagnoses used to create a collective differential. For the weighting rule, we looked at 3 other approaches: 1/*n*^2^ (which heavily downweights diagnoses beyond the first 2), equal weights for all diagnoses (which does not downweight lower-ranked diagnoses), or equal weights with a randomly ordered 5 diagnoses for each user (which ignores ranking). For the number of diagnoses in the collective differential, we looked at performance using the top 1 through 5 diagnoses. For user diagnoses, we used the top 5 user diagnoses with the 1/*n* weighting rule to create a collective differential. The base case uses the top 3 diagnoses for both the collective differential and user diagnoses.

### Statistical Analysis

Individual diagnostic accuracy was calculated by randomly selecting 1 solve attempt from the 10 solve attempts sampled for each case, noting whether it was correct or incorrect, and then calculating the average for the 1572 cases. To calculate group diagnostic accuracy, we first created groups of *x* users (*x* = 2 to 9) among the remaining 9 solve attempts for each case, 1 group for each size from 2 through 9 per case. The overall diagnostic accuracy for groups of *x* users was defined as the proportion of correct collective differentials among all groupings of *x* users (ie, the proportion of correct solves for all groups of 4 users).

To test for differences in accuracy between individuals and groups within the same specification rules, we used a variation of the McNemar test to account for pairing of data by case (ie, for each case, we have an individual and a group answer). The standard McNemar test assumes independence between each case solution, when in fact some individual users participated in multiple case solutions. To address this, we calculated the test statistic for the McNemar test using hierarchical logistic regression with random effects for each individual user to account for dependence of solutions within individual users. Differences in accuracy between groups across different rule specifications were tested using a *z *test for proportions. All statistical analyses were performed in R version 3.5.0 (R Project for Statistical Computing), with 2-sided *P* < .05 considered significant.^17,18^

Because the users in our study sample had a variety of training backgrounds, we evaluated whether a group’s performance varied based on the training level of the collective groups. There were too few medical students or attending physicians to create collective differentials across the 1572 cases; therefore, we examined the performance of groups in each size category with no medical students or attending physicians. We also evaluated whether group and individual diagnostic accuracy varied by presenting symptom. We identified the most common presenting symptoms with at least 50 cases in our sample (chest pain, shortness of breath, abdominal pain, and fever) and computed individual and group diagnostic accuracy for the subset of relevant cases as described above.

Finally, we compared the diagnostic accuracy of groups of users to individual subspecialists on subspecialty cases. We identified all subspecialty cases solved by the corresponding subspecialists (eg, cardiology cases solved by cardiologists) and chose a single, corresponding subspecialist among the 10 solve attempts. The individual subspecialist diagnostic accuracy was computed as the average across the subspecialty cases, with group diagnostic accuracy calculated excluding the individual subspecialist from each group. When more than 1 subspecialist solved a single subspecialty case (eg, 2 nephrologists solved 1 nephrology case), only 1 subspecialist was used to compute the individual subspecialist accuracy.

## Results

### User Characteristics and Individual Accuracy

Of the 2069 users solving 1572 cases from the Human Dx data set, 1452 (70.2%) were trained in internal medicine, 1228 (59.4%) were residents or fellows, 431 (20.8%) were attending physicians, and 410 (19.8%) were medical students. Among the users, 207 (10.0%) were from surgery and other specialties (Table). Each specialty participated in the system roughly proportional to their representation in the sample (eg, internal medicine physicians were 70.2% of the users, and they solved 65.8% of cases). There was a wide distribution in the number of cases solved by individual users in our data set, with 748 users (36.2%) solving only 1 case but 580 (28.0%) solving 5 or more cases. Twenty-two users (1.1%) in our data set solved more than 100 cases each.

**Table.** User Characteristics of the Study Sample^a^

User Characteristic	No. (%) (N = 2069)
Training level	
Attending physician	431 (20.8)
Fellow or resident	1228 (59.4)
Medical student	410 (19.8)
Specialty	
Internal medicine	1452 (70.2)
General	1378 (66.6)
Subspecialty	74 (3.6)
Surgery	51 (2.5)
Other specialties	156 (7.5)
Medical student	410 (19.8)
Geographic location	
United States	1881 (90.9)
Other than the United States	188 (9.1)

^a^ All physicians and medical students using the Human Diagnosis Project (Human Dx) platform for the sample of 1572 cases used in the present analysis.

Using the top 3 diagnoses given by each user to adjudicate solutions, the diagnostic accuracy of all users was 62.5% (95% CI, 60.1%-64.9%). The accuracy of individual residents and fellows was 65.5% (95% CI, 63.1%-67.8%) compared with 55.8% for medical students (95% CI, 53.4%-58.3%; *P* < .001 for difference vs residents and fellows by *z* test for proportions) and 63.9% for attending physicians (95% CI, 61.6%-66.3%; *P* = .10 for difference vs residents and fellows).

### Overall Diagnostic Accuracy of Collective Differentials

**Figure 1.**
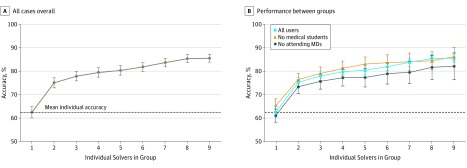
Diagnostic Accuracy for the Base Case Rule by Group Size Overall and by User Training Each point shows the accuracy of groups using the base case rule of using all diagnoses from each to make a collective differential diagnosis list and assessing the top 3 weighted diagnoses in the collective for diagnostic success. A, Curve for all cases overall. B, Comparison of performance between groups with and without medical students or attending physicians. To create the collective differential diagnosis list, individuals’ diagnoses were weighted inversely to their position on a differential list (eg, the first diagnosis has a weight of 1, the second is 1/2, and so on). For each group, we summed diagnosis weights to produce a ranked list and used the top 3 diagnoses in this collective differential to assess accuracy. The y-axis represents accuracy, the percentage of cases with the correct diagnosis in the top 3 collective diagnoses listed, for increasing group sizes of randomly chosen users. The dashed line indicates the average accuracy for individual solvers across the 1572-case sample. Error bars indicate 95% CIs.

We compared the diagnostic accuracy of groups using the base case 1/*n* proportional weighting rule vs the mean accuracy by an individual (Figure 1). With increasing group size, collective intelligence was associated with greater diagnostic accuracy than mean individual accuracy: 85.6% accuracy (95% CI, 83.9%-87.4%) for group of 9 vs 62.5% accuracy (95% CI, 60.1%-64.9%) for individuals (difference, 23.0%; 95% CI, 14.9%-31.2%; *P* < .001 by the McNemar test). Increasing diagnostic accuracy was associated with increased group size (eg, 12.5% difference vs individual for groups of 2 [95% CI, 9.3-15.8; *P* < .001] and 17.8% difference vs individual for groups of 5 [95% CI, 14.0%-21.6%; *P* < .001] by the McNemar test). Diagnostic accuracy was similar for groups of 9 across users, groups of 9 without medical students (86.2% accuracy; 95% CI, 82.2%-90.2%), and groups of 9 without attending physicians (82.1% accuracy; 95% CI, 76.4%-84.8%; *P* > .26 for all 3 pairwise comparisons between groups in Figure 1B by *z* test for proportions).

**Figure 2.**
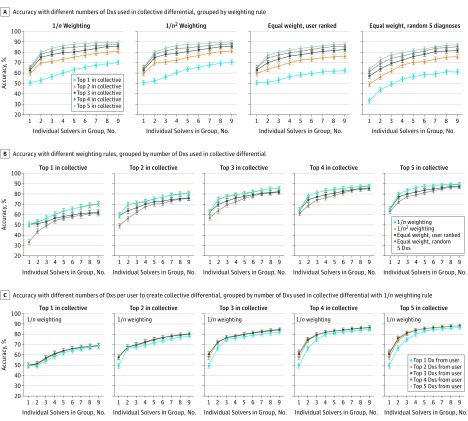
Range of Diagnostic Accuracy of Collective Differential Diagnoses (Dxs) Across Different Specifications Each panel shows the accuracy of groups using multiple variations on the rules for making a collective differential and adjudicating a solution. The base case used in the other figures corresponds to 1/*n* weighting with 3 Dxs used in the collective differential. In all panels, the y-axis represents accuracy, the percentage of 1572 cases with the correct Dx in the collective differential, for increasing group sizes of randomly chosen physician users. Error bars indicate 95% CIs. A, A different weighting scheme was applied to the *n*th Dx for each user in the group: 1/*n*, 1/*n*^2^, equal weight to all Dxs, and equal weight for 5 randomly ordered Dxs per user. A separate colored curve is shown for the change in accuracy using the top 1 through 5 Dxs in the collective differential to adjudicate a solution. B, The same results as in part A, but each panel instead shows 4 separate colored curves for each weighting scheme using the top 1 through 5 Dxs in the collective differential to adjudicate a solution. C, The 1/*n* weighting rule, with each panel showing 5 separate curves for using the top 1 through 5 Dxs from each user’s solution to create the collective differential.

The change in diagnostic accuracy from individual to larger groups varied across different specifications for constructing the collective differential and assessing success, but groups were consistently associated with higher performance than individuals. Using more diagnoses in the collective differential to adjudicate success was predictably associated with greater group accuracy. Accuracy for a group of 9 for the 1/*n* weighting rule using 1 diagnosis in the collective differential was 70.3% (95% CI, 68.0%-72.6%) and accuracy for a group of 9 for the 1/*n* weighting rule using 5 diagnoses in the collective differential was 89.6% (95% CI, 88.1%-91.1%) (*P* < .001 by *z* test for proportions) (Figure 2).

Across different weighting schemes, 1/*n* and 1/*n*^2^ had nearly equivalent performance. For example, in the base case of using the top 3 diagnoses in the collective differential, groups of 9 were associated with 85.6% accuracy for 1/*n* (95% CI, 83.9%-87.4%) vs 85.8% accuracy for 1/*n*^2^ (95% CI, 84.0%-87.5%; *P* = .95 for difference with 1/*n* by *z* test for proportions) (Figure 2). Weighting schemes that ignored the rank ordering of diagnoses by users (equal weight for all diagnoses or random 5 diagnoses per user) had lower associated accuracy than the other weighting schemes, particularly with few diagnoses considered by the collective differential. For example, groups of 9 were associated with 62.3% accuracy for the equal weight (95% CI, 59.9%-64.7%) vs 70.3% accuracy for 1/*n* (95% CI, 68.0%-72.6%) using only 1 diagnosis in the collective differential (*P* < .001 for difference with 1/*n* by *z* test for proportions).

The number of user diagnoses used to construct the collective differential made little difference in the accuracy beyond the top 2 (shown in Figure 2 using the 1/*n* rule). Group accuracy still outperformed individual diagnosis in the most restrictive definition, using only 1 diagnosis per user and only the top 1 diagnosis in the collective differential (absolute difference vs individual for group of 9 by McNemar test, 19.7%; 95% CI, 12.7%-26.7%; *P* < .001).

### Diagnostic Accuracy of Collective Intelligence in Subgroups of Cases

**Figure 3.**
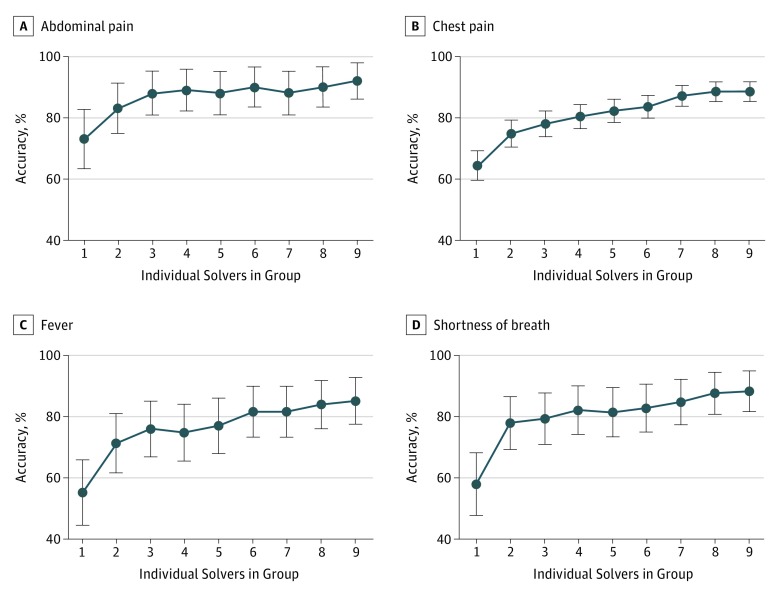
Diagnostic Accuracy and Group Size of Physicians by Presenting Symptom Shown is the accuracy, the percentage of cases with the correct diagnosis in the top 3 diagnoses listed, for increasing group sizes of randomly chosen physician users across 4 subgroups of cases with the most prevalent presenting symptoms. The presenting symptoms are abdominal pain (81 cases), chest pain (380 cases), fever (84 cases), and shortness of breath (90 cases). Error bars indicate 95% CIs.

We analyzed the association of collective differential groups for cases with 4 presenting symptoms: chest pain, shortness of breath, abdominal pain, and fever. When we used the base case set of rules, collective intelligence from increasing group size was associated with improved diagnostic accuracy across all groups of cases (Figure 3). Absolute improvement in group diagnostic accuracy from individuals to groups of 9 ranged from 17.3% (95% CI, 6.4%-28.2%; *P* = .002 by the McNemar test) for abdominal pain to 29.8% (95% CI, 3.7%-55.8%; *P* = .02) for fever.

**Figure 4.**
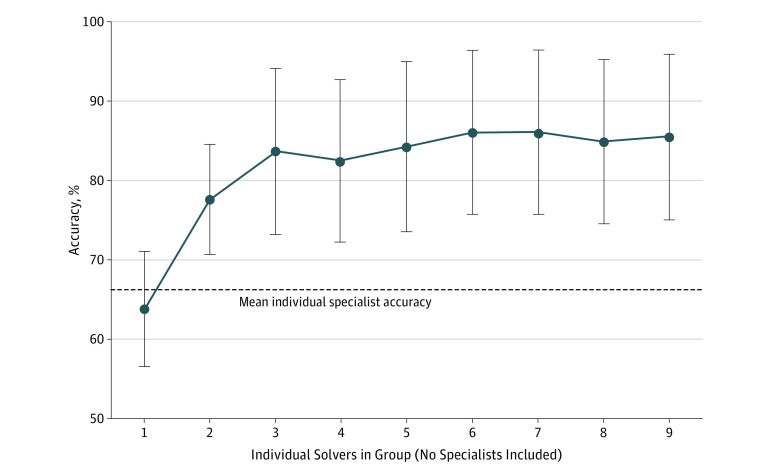
Diagnostic Accuracy for Nonspecialist Groups Compared With Individual Specialists Shown is the accuracy, the percentage of cases with the correct diagnosis in the top 3 diagnoses listed, for increasing group sizes of randomly chosen nonspecialist physician users. Each group is composed of a set of users whose specialty does not match the specialty of the individual case. The dashed line indicates the individual accuracy of specialist physicians whose specialty matches the case (eg, cardiology for a cardiovascular presenting symptom). Error bars indicate 95% CIs.

We also compared the diagnostic accuracy of groups with that of the individual specialist on cases in their respective specialty (Figure 4). Overall, 54 specialists solved 166 cases with diagnostic accuracy of 66.3% (95% CI, 59.1%-73.5%), compared with nonmatched specialty accuracy of 63.9% (95% CI, 56.6%-71.2%). Groups outperformed individual specialists in their specialty for all group sizes (accuracy, 77.7%; 95% CI, 70.1%-84.6%; *P* < .001 vs individuals by McNemar test for a group of 2 to 85.5%; 95% CI, 75.1%-95.9%; *P* < .001 vs individuals for a group of 9).

## Discussion

Collective intelligence with groups of physicians and trainees was associated with improved diagnostic accuracy compared with individual users across multiple clinical disciplines. To our knowledge, this study is the largest investigation to date of collective intelligence for general clinical diagnosis both by number of physicians and number of cases. Diagnostic accuracy was consistently greater with increasing group size, similar to findings of earlier literature on medical student teams^11,16^ and combinations of user opinions for image interpretation.^8,9,10^ These findings held across a broad range of specifications for creating a collective differential. These findings suggest that using the concept of collective intelligence to pool many physicians’ diagnoses could be a scalable approach to improve diagnostic accuracy.

Previous literature comparing individual with collaborative diagnosis is limited and has focused primarily on consensus-driven methods that require a number of factors to foster collaboration among groups.^5,19^ Collective intelligence and the associated improvement in accuracy, however, could also arise from simply combining independent assessments of a case without the time and coordination required for group discussions. Although collective intelligence is a relatively new concept, the medical profession has long used collaborative decision making processes such as the Delphi technique and the nominal group technique,^20,21^ as well as less formal processes such as case conferences.

The optimal approach to creating a collective differential may depend on the use case. If finding a single diagnosis with the highest statistical accuracy is necessary, it may be optimal to limit the collective differential to 1 or 2 diagnoses. On the other hand, if a broader list for further diagnostic workup is clinically appropriate, a longer list could be helpful. Regardless of the number of collective diagnoses considered, the consistently higher performance of weighting rules that de-emphasized diagnoses with lower ranks implies that diagnosis rank is important to build into a collective intelligence approach. This is also reflected by the minimal change in accuracy beyond using the top 2 diagnoses from users for constructing collective differentials. Further research is needed to explore optimal rules for combining user diagnoses in different clinical scenarios.

Collective intelligence could be adapted to a wide range of clinical settings. In resource-rich settings, the modern proliferation of smartphones and widespread use of the internet can potentially enable near real-time gathering and pooling of group decisions. However, collective intelligence need not be facilitated by software; it is possible that even with paper and pencil, diagnostic accuracy could be improved with a collective intelligence approach. If this phenomenon is replicable outside of a digital context, it implies that collective intelligence could be broadly applicable, including in low-resource settings. However, the additional benefit of using the collective intelligence approach would need to be weighed against the time and workload necessary to implement in practice. Further evaluation of this diagnosis approach in a real-world setting is necessary to assess this, especially given the potential complexity of implementation in clinical settings.

We found that groups of all sizes outperformed individual subspecialists on cases in their own subspecialty. This may not be surprising, given recent data suggesting that final diagnoses differed from the referral diagnosis in 21% of subspecialist referrals; for most cases, diagnoses were unchanged or only more refined.^22^ Given the mismatch between supply and demand for subspecialty services for low-income patients,^23,24^ a collective intelligence approach could provide valuable diagnostic assistance for primary care clinicians in areas that struggle with human capital in health care and have higher rates of diagnostic error.^25^

### Limitations

Our study has several important limitations. First, users who contribute to Human Dx may not be a representative sample of the medical community and therefore differ in diagnostic accuracy. To our knowledge, our data set includes the largest number of attending physicians (431) to date in a study of collective intelligence; nonetheless, trainees composed nearly 80% of the population. However, the diagnostic error rate in our data set is within the range identified in prior studies of diagnostic error.^5,6,26^ Second, Human Dx was not designed specifically to assess collective intelligence. It is possible that a platform to explicitly generate collective diagnoses would have different performance than we observed. Another pair of limitations are the potential for inaccurate assessment of diagnostic solutions by manual review and misattribution of specialty labels associated with each case. These 2 issues could create bias in our assessment of diagnostic solutions and the specialty match between subspecialists and case specialty, although we do not believe these issues should be associated with differences in the relative performance between individuals vs groups. Also, we were not able to assess the extent to which greater accuracy would also have been associated with changes in patient treatment. This will be an important question for future work. The breadth of the cases may not be representative of types of cases likely to be encountered in practice or those that are most amenable to performance improvement with collective intelligence.

## Conclusions

We found that collective diagnosis by groups was associated with improved accuracy over individual diagnosis. Given the few proven strategies to address the high prevalence of misdiagnosis, these results suggest that collective intelligence merits further study in a real-world clinical setting.
